# Evaluación de siete programas bioinformáticos para el análisis terciario de datos genómicos generados a partir de la secuenciación del exoma completo en un grupo piloto de pacientes

**DOI:** 10.1515/almed-2024-0101

**Published:** 2025-02-10

**Authors:** Nerea Bastida-Lertxundi, Itxaso Martí-Carrera, Borja Laña-Ruíz, Otilia Martínez-Múgica Barbosa, Raquel Muguerza-Iraola, Raquel Sáez-Villaverde, Julien S. Crettaz

**Affiliations:** Instituto de Investigación Sanitaria Biogipuzkoa, Grupo de Investigación de Neurogenética, biología y terapias de ARN – NeuroRNA, San Sebastián, España; Osakidetza, Organización Sanitaria Integrada Donostialdea, Unidad de Genética Clínica, Hospital Universitario Donostia, San Sebastián, España; Instituto de Investigación Sanitaria Biogipuzkoa, Grupo de Investigación de Pediatría, San Sebastián, España; Osakidetza, Organización Sanitaria Integrada Donostialdea, Servicio de Pediatría, Hospital Universitario Donostia, San Sebastián, España; Departamento de Pediatría, Universidad del País Vasco UPV/EHU, San Sebastián, España

**Keywords:** priorización automática, clasificación automática, inteligencia artificial, exoma completo, análisis terciario, programa bioinformático

## Abstract

**Objetivos:**

Evaluar siete programas bioinformáticos de priorización y clasificación automática que utilizan algoritmos de inteligencia artificial.

**Métodos:**

Se evaluaron 24 variantes genéticas que explicaran el fenotipo de 20 pacientes. Los archivos FASTQ se cargaron paralelamente en los siguientes programas bioinformáticos: Emedgene, eVai, Varsome Clinical, CentoCloud, y QIAGEN Clinical Insight (QCI) Interpret, SeqOne y Franklin. Para la priorización y clasificación automática, se utilizó el fenotipo de los pacientes, introduciéndolo en los programas mediante términos HPO. La clasificación de referencia se estableció siguiendo los criterios y recomendaciones de las guías clínicas de la American College of Medical Genetics (ACMG) and Genomics, Association of Molecular Pathology y ACMG/ClinGen.

**Resultados:**

SeqOne tuvo el mejor rendimiento en priorización, colocando 19 de 24 variantes en el Top 1, cuatro en el Top 5 y una en el Top 15, seguido por CentoCloud y Franklin. QCI Interpret no priorizó seis variantes y no detectó una, Emedgene no priorizó una y no detectó otra, y Varsome Clinical no priorizó cuatro variantes. Franklin clasificó correctamente el 75 % de las variantes evaluadas, seguido por Varsome Clinical (67 %) y QCI Interpret (63 %).

**Conclusiones:**

Respecto a la priorización automática, tanto SeqOne, CentoCloud, como Franklin realizaron una priorización automática de calidad, priorizando todas las variantes. En cuanto a la clasificación automática, Franklin mostró mayor concordancia con la referencia y menos discordancias con implicación clínica. Como conclusión final, Franklin parece ser actualmente el programa con mejor rendimiento global, pero se requieren más estudios para confirmar estos resultados.

## Introducción

Se estima que hasta el 5,9 % de la población padece una enfermedad rara y hasta el 50 % de estos pacientes nunca logran obtener un diagnóstico concreto [1], 2]. Aquellos pacientes cuyo diagnóstico es incierto, suelen embarcarse en lo que se conoce como la odisea diagnóstica, un proceso que implica consultas con múltiples especialistas, exámenes de imágenes médicas y una infinidad de análisis de laboratorio clínico [3]. Proporcionar un diagnóstico molecular mejora el manejo de la enfermedad, optimiza los tratamientos y la vigilancia, y permite realizar un asesoramiento genético específico para el paciente y la familia en cuanto a complicaciones, riesgo de recurrencia y opciones reproductivas [4].

La secuenciación del exoma completo (WES del inglés whole exome sequencing) ha revolucionado el diagnóstico genético al permitir la secuenciación en paralelo de aproximadamente 22.000 genes codificantes, que representan cerca del 2 % del genoma y donde se estima que se encuentran el 85 % de las variantes causantes de enfermedad [5], 6]. Tras superar la revolución tecnológica de la secuenciación masiva las plataformas de alta procesabilidad permiten en cuestión de horas secuenciar varios exomas a un coste razonable y con gran exactitud [7]. Actualmente el sector se encuentra inmerso en la era de la gestión de los datos genómicos, por un lado, su almacenamiento puede llegar a ser un reto debido a las exigencias de seguridad, privacidad y trazabilidad y por el otro, hallar la causa genética puede asemejarse a buscar una aguja en un pajar [8], 9]. La WES de un individuo contiene de media unas 100.000 variantes. En general, la tarea de filtrado, priorización, interpretación y clasificación de las variantes clínicamente relevantes es la etapa más desafiante del diagnóstico genético en entornos clínicos y el cuello de botella a la hora de realizar este tipo de estudios genéticos. Los datos generados por las plataformas de secuenciación requieren de unos pasos de procesamiento bioinformáticos previos a poder comenzar a analizar el listado de variantes candidatas. En entornos asistenciales, los profesionales clínicos se apoyan en programas comerciales para realizar este procesamiento. Estos programas llevan a cabo un análisis secundario de los datos genómicos, donde se identifican las variantes discordantes en comparación con el genoma de referencia [10], 11]. Además, realizan un análisis terciario que consiste en anotar cada variante, recopilando información relevante tanto intrínseca a la variante como aquella disponible en bases de datos y en la literatura científica [12], [13], [14], [15], [16], [17]. Posteriormente, se procede al filtrado y priorización de las variantes.

Hasta hace poco, este último paso requería una revisión manual de un listado de variantes en tablas, utilizando filtros de forma bastante básica. Además, era necesario clasificar las variantes manualmente, siguiendo las recomendaciones de las guías clínicas [18], 19]. Todo esto hacía que la identificación de la variante causal fuera un proceso complicado y laborioso.

Para optimizar el análisis terciario, los programas han comenzado a permitir la introducción de datos de los pacientes, como la edad, el género, el fenotipo y el inicio de los síntomas, entre otros aspectos. Y es aquí donde toman especial relevancia los términos HPO (del inglés human phenotype ontology) una iniciativa enfocada en la estandarización de los fenotipos de los pacientes. Esta normalización posibilita que los programas puedan crear interrelaciones entre los términos HPO del paciente, los genes asociados a estos términos, y las patologías descritas junto a otros parámetros anteriormente mencionados durante el proceso de anotación [20].

Actualmente, la gran novedad de estos programas bioinformáticos es que incorporan algoritmos de inteligencia artificial (IA) de aprendizaje automático, los cuales crean redes neuronales artificiales. Estas redes son capaces de correlacionar diversos parámetros, como el genotipo, los genes, las patologías, las bases de datos poblacionales, las bases de datos de asociación entre variantes y fenotipos, así como los predictores y la sintomatología de los pacientes, además de las publicaciones científicas, entre otros. Estas redes posibilitan realizar una priorización de variantes de forma automática, eficaz y rápida. Además, estos programas tienen la capacidad de integrar las recomendaciones de las guías clínicas de la American College of Medical Genetics and Genomics (ACMG)/Association of Molecular Pathology (AMP) así como las de ACMG/ClinGen [18], 19]. Esto permite no solo una priorización automática, sino también una clasificación automática de las variantes.

Se cree que la aplicación de la IA en la medicina genómica potenciará la implementación del WES, entre otras aplicaciones genómicas, en la práctica clínica, haciendo que estén al alcance de todos los pacientes de forma rutinaria. Este avance, sin duda, facilitará la transformación de la asistencia sanitaria [21].

Hasta la fecha, se ha publicado un número reducido de artículos que comparan programas de priorización de variantes. La mayoría evalúan únicamente programas de acceso libre y utilizan una aproximación de filtrado y priorización basados en el fenotipo de los pacientes. Además, solo uno de ellos incluye algoritmos de IA [22], [23], [24], [25], [26], [27], [28], [29].

Este estudio tiene como objetivo llevar a cabo una evaluación objetiva e independiente de diversos programas bioinformáticos disponibles en el mercado para la interpretación de datos genómicos.

El objetivo final de este proyecto es implementar el WES a nivel asistencial. Para ello, es necesario disponer de datos objetivos que permitan seleccionar el programa más idóneo. En definitiva, contar con una capacidad de priorización y clasificación automática eficaz permitirá a los profesionales del laboratorio clínico reducir el tiempo requerido para el análisis y sus costes asociados, mejorar los tiempos de respuesta, aumentar la tasa diagnóstica y hacer frente al continuo aumento de demanda de este tipo de estudios genómicos [30], 31].

## Materiales y métodos

Se seleccionaron retrospectivamente 20 pacientes de forma aleatoria que presentaban una o varias variantes relacionadas con su fenotipo, a partir de un estudio de WES. Estos pacientes fueron atendidos por diversas especialidades en el Hospital Universitario Donostia.

La selección de estos pacientes se realizó con el objetivo de incluir la mayor gama de variantes deletéreas y de tipos de herencias. Se incluyeron 24 variantes genéticas, las cuales fueron clasificadas por el laboratorio externo y fueron revisadas por los profesionales de la Unidad de Genética Clínica del Hospital Universitario Donostia (UGC-HUD) determinando la clasificación asignada como de referencia (véase Material Suplementario, Tabla 1 y Tabla 2). La clasificación de referencia se estableció siguiendo los criterios y recomendaciones de las guías clínicas de la ACMG/AMP y ACMG/ClinGen [18], 19].

Así se incluyeron seis cambios de número de copia (CNV del inglés copy number variation) con tamaños comprendidos entre 1,9 kb y 9,4 Mb, tanto deleciones como duplicaciones, todos de herencia autosómica dominante excepto una deleción ligada al cromosoma X (véase Material Suplementario, Tabla 3). En cuanto a los cambios de un único nucleótido (SNV del inglés single nucleotide variation) se incluyeron 12, entre los cuales se encontraban cinco variantes de cambio de sentido (missense), cuatro sin sentido (nonsense), dos variantes en sitios de splicing canónicos y una variante sinónima (silent). También se incluyeron cinco pequeñas deleciones y una pequeña duplicación que alteraban el marco de lectura (frameshift). La herencia de estas 18 variantes era heterogénea (véase Material Suplementario, Tabla 4). Las variantes a estudio estaban clasificadas como patogénica clase 5 o como probablemente patogénica clase 4.

Los datos obtenidos tras la secuenciación de cada paciente en formato FASTAQ fueron cargados de forma paralela para la ejecución del análisis secundario y terciario de los datos genómicos en siete programas bioinformáticos. Tras analizar la oferta disponible se seleccionaron los siguientes siete programas bioinformáticos por ser líderes en el sector y por tener implementados algoritmos de IA: Emedgene (Illumina^®^, Inc, CA, EE.UU), eVai (enGenome, Pavia, Italia), Varsome Clinical (Saphetor SA, Lausanne, Suiza), CentoCloud^®^ (Centogene GmbH, Rostock, Alemania), CLC genomics workbench para el análisis secundario y QIAGEN Clinical Insight (QCI) Interpret para el terciario (Qiagen GmbH, Hilden, Alemania), SeqOne (Montpellier, Francia) y Franklin (Genoox, Tel Aviv-Yafo, Israel). Además de los datos genómicos de cada paciente, se integró el fenotipo empleando los mismos términos HPO en el análisis bioinformático realizado en cada plataforma. (véase Material Suplementario, Tabla 5).

Primero, se evaluó la capacidad de priorizar variantes de cada programa. Se analizó en qué posición colocaba cada programa las 24 variantes causales. Cuando la variante se encontraba en primera posición se definió que se encontraba en el Top 1. Cuando se encontraba en las cinco primeras posiciones era Top 5, en las diez primeras Top 10 y en las 15 primeras Top 15. Cuando la variante se encontraba más allá de la posición 16 se determinó que no fue priorizada (NP) y cuando la variante causal no se encontraba en el listado total de variantes se consideró que no fue detectada (ND). Además, se agruparon las variantes NP y ND bajo el concepto de “incidencia” para definir un fallo de selección que puede llevar claramente a un no-diagnóstico. En segundo lugar, se llevó a cabo una comparación de la clasificación automática realizada por los programas bioinformáticos. Todos los programas incluidos en este estudio utilizan un enfoque de clasificación automática que sigue las recomendaciones establecidas en las guías clínicas de la ACMG/AMP y ACMG/ClinGen [18], 19]. Esta clasificación automática de las 24 variantes se contrastó con la clasificación de referencia previamente asignada. Debido a que todas las variantes estudiadas eran patogénicas y probablemente patogénicas, se consideró discordancia sin implicación clínica cuando una variante era clasificada como patogénica en vez de probablemente patogénica y viceversa. En cambio, se consideró una discordancia con implicación clínica cuando la variante fue clasificada como de significado incierto y benigna.

## Resultados

### Evaluación de la priorización automática

**Figura 1:**
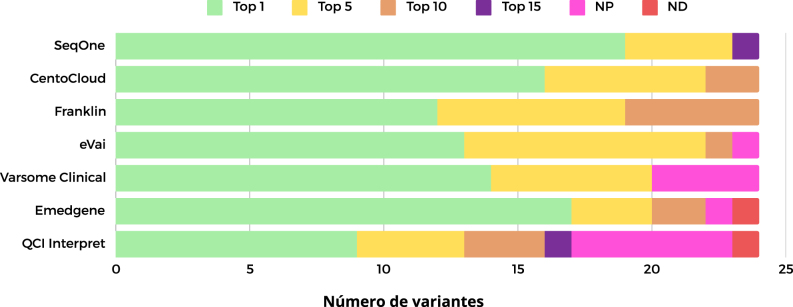
Ranking de programas por la capacidad de priorización de las variantes evaluadas. NP, no priorizado; ND, no detectado; QCI Interpret, Qiagen clinical Insight Interpret.

Los mejores programas, considerando las posiciones Top 1 y Top 5, fueron SeqOne, CentoCloud y eVai. Si se toma en cuenta el Top 10, los dos programas más destacados fueron CentoCloud y Franklin (Figura 1).

**Figura 2:**
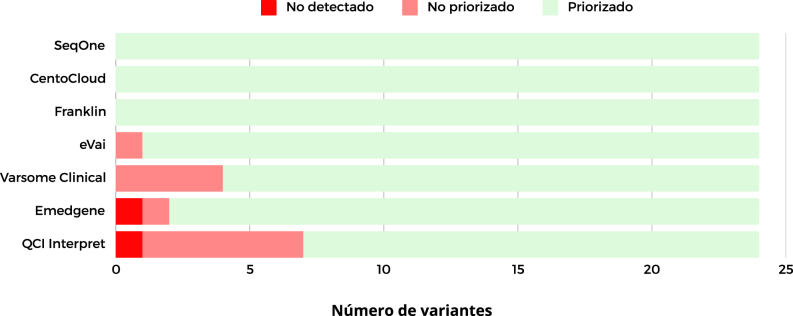
Ranking de programas por número de variantes sin detectar y sin priorizar. QCI Interpret, Qiagen clinical Insight Interpret.

Emedgene dispuso 22 variantes, lo que representa el 92 % de las mismas, dentro del Top 10. Sin embargo, no priorizó una SNV nonsense en el gen *PAX9* del paciente R14 y no detectó una CNV del paciente R1 (véase Material Suplementario, Tabla 6). Varsome Clinical no priorizó cuatro variantes (17 %), de las cuales tres eran CNVs, pero el resto de las variantes se localizaron dentro de las posiciones Top 1, 14 (58 %) y Top 5, seis (25 %). QCI Interpret dio los peores resultados, tuvo el mayor número de variantes no priorizadas, seis (25 %) y no fue capaz de detectar una SNV (4 %) en el gen *CDKL5* en el paciente R15 (véase Material Suplementario, Tabla 7). En el ranking de programas por número de incidencias, QCI Interpret ocupó el último lugar, registrando seis variantes no priorizadas y una no detectada. En sexto lugar, se posicionó Emedgene, que no logró detectar una variante y tampoco priorizó otra. Varsome Clinical se ubicó en el quinto puesto, sin poder priorizar cuatro variantes. Finalmente, eVai quedó en tercera posición, ya que no priorizó una variante (Figura 2).

En total, hubo 14 incidencias. Emedgene no detectó la CNV 2p16.3 (DEL) y QCI Interpret no detectó la variante *CDKL5*:c.283-2A>G. Varsome Clinical mostró dificultad para priorizar CNVs no priorizando tres de las seis evaluadas. Y QCI Interpret no priorizó cinco variantes de las cuales dos eran missense, dos frameshift y una nonsense (Tabla 1).

**Tabla 1:** Variantes no priorizadas y no detectadas.

Paciente	Localización Cromosómica	Tipo de CNV	Incidencia	Software
R1	2p16.3	Del	ND	Emed
			NP	Varso, QCII
R4	15q11.2	Del	NP	Varso
R9	Xp22.31	Del	NP	Varso

**Paciente**	**Gen**	**Variante**	**Incidencia**	**Software**

R15	*CDKL5*	c.283-2A>G	ND	QCII
R1	*SPAST*	c.1617-2A>G	NP	Varso
R8	*EXT1*	c.1037G>T	NP	QCII
R12	*CNOT1*	c.2071del	NP	QCII
R14	*PAX9*	c.554C>A	NP	QCII, Emed
R17	*BSND*	c.23G>A	NP	eVai, QCII
R19	*JAG1*	c.221_224del	NP	QCII

CNV, cambio de número de copias; NP, no priorizado; ND, no detectado; Emed, Emedgene; QCII, Qiagen Clinical Insight Interpret; Varso, Varsome Clinical.

A nivel global, y excluyendo los programas que presentan variantes no priorizadas y no detectadas debido a su significativo impacto clínico, el programa SeqOne destacó por su rendimiento, logrando colocar 19 variantes en primer lugar, cuatro en el Top 5 y una en el Top 15. En segundo lugar, se posicionó CentoCloud, con 16 variantes en el Top 1, seis en el Top 5 y dos en el Top 10. Finalmente, Franklin ocupó la tercera posición, con 12 variantes en el Top 1, siete en el Top 5 y cinco en el Top 10.

### Evaluación de la clasificación automática

**Figura 3:**
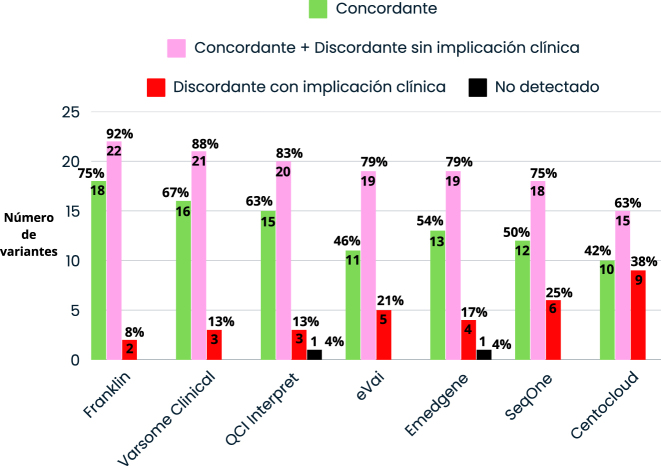
Comparativa de la clasificación automática con la de referencia de cada programa. QCI Interpret: Qiagen clinical Insight Interpret.

En general todos los programas realizaron una clasificación automática de calidad utilizando las reglas de las guías clínicas [18], 19]. Franklin coincidió exactamente en la clasificación automática asignada con la de referencia en 18 de las 24 variantes (75 %), seguido de Varsome Clinical en 16 (67 %), QCI Interpret en 15 (63 %), Emedgene en 13 (54 %), SeqOne en 12 variantes (50 %), eVai en 11 variantes (46 %) y CentoCloud en 10 (42 %). Franklin realizó una clasificación adecuada (concordante con la clasificación de referencia más las discordantes sin implicación clínica) en 22 de las 24 variantes (92 %), seguido de Varsome Clinical en 21 variantes (88 %), QCI Interpret en 20 variantes (83 %), Emedgene y eVai en 19 variantes (79 %), SeqOne en 18 variantes (75 %) y CentoCloud en 15 variantes (63 %). Respecto a los programas con mayor número de clasificación discordante con implicación clínica, CentoCloud quedó peor posicionada ya que a nueve de las 24 (37,5 %) de las variantes se le asignó una clasificación discordante con implicación clínica, ocho de las cuales se clasificaron como de significado incierto y una se clasificó como benigna. En cambio, Franklin tuvo el menor número de clasificaciones discordantes con implicación clínica, dos de las 24 evaluadas (8,3 %) (Figura 3).

**Figura 4:**
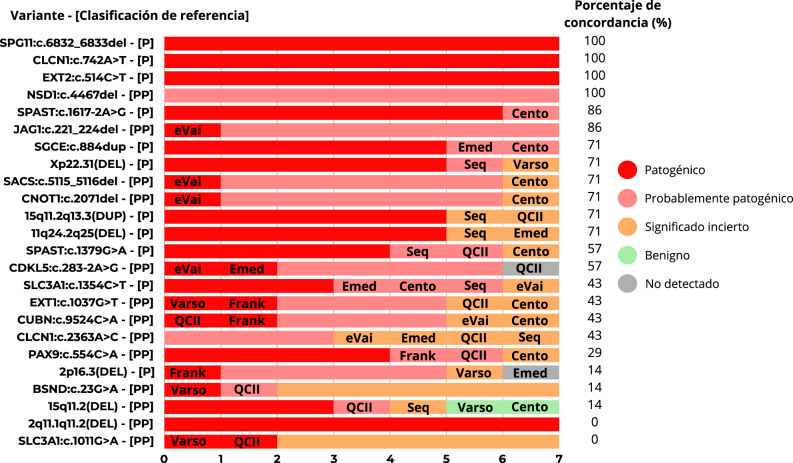
Comparativa de la clasificación automática de los programas por cada variante, concordancia con la clasificación de referencia y programas con discordancia. Cento, CentoCloud; Emed, Emedgene; Frank, Franklin; QCII, Qiagen clinical Insight Interpret; Seq, SeqOne; Varso, Varsome clinical.

Inspeccionando las variantes individualmente, solo en cuatro de las variantes (SPG*11*:c.6832_6833del, *CLCN1*:c.742A>T, *EXT2*:c.514C>T y *NSD1*:c.4467del) todos los programas estuvieron de acuerdo en cuanto a su clasificación y fueron concordantes con la clasificación de referencia. En las variantes *SPAST*:c.1617-2A>G, *JAG1*:c.221_224del hubo un programa y en las variantes *SGCE*:c.884dup y *CDKL5*:c.283-2A>G hubo dos programas que tuvieron discrepancia sin implicación clínica. Al respecto de la variante 2q11.1q11.2(DEL) con clasificación de referencia como probablemente patogénica, todos los programas concordaron y la clasificaron como patogénica. En otras 15 variantes, se detectó una discrepancia con implicación clínica. Por último, en la CNV 15q11.2(DEL) clasificada como probablemente patogénica por UGC-HUD y por QCI Interpret, hubo tres programas Emedgene, eVai y Franklin que la clasificaron como patogénica, SeqOne como de significado incierto y hubo dos, CentoCloud y Varsome Clinical que la clasificaron como benigna de clase 1 (Figura 4).

**Figura 5:**
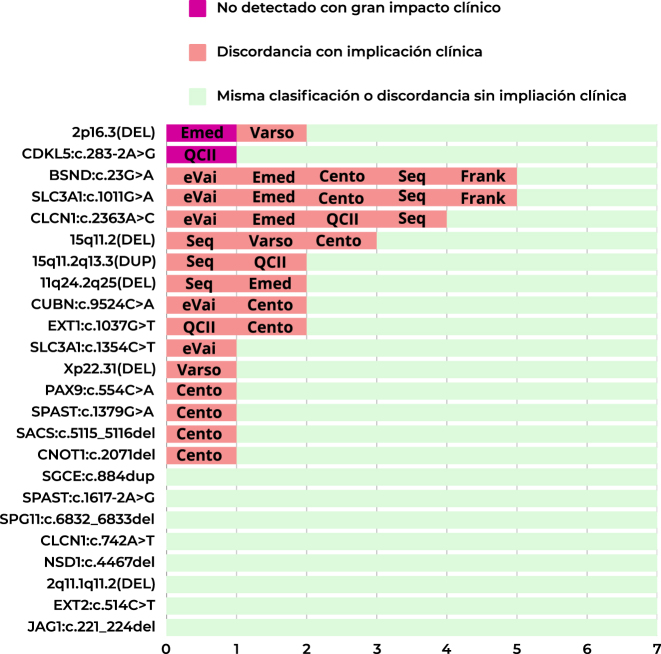
Concordancias y discordancias en la clasificación automática respecto a la clasificación de referencia y los programas implicados. Cento, CentoCloud; Emed, Emedgene; Frank, Franklin; QCII, Qiagen clinical Insight Interpret; Seq, SeqOne; Varso, Varsome clinical.

En total, se observaron 32 discordancias en la clasificación que pudieran tener implicación clínica en cuanto al correcto diagnóstico de los pacientes. Para las variantes *BSND*:c.23G>A, *SLC3A1*:c.1011G>A, *CLCN1*:c.2363A>C y 15q11.2(DEL) tres o más de tres programas asignaron una clasificación discordante con implicaciones clínicas (Figura 5).

## Discusión

Los avances tecnológicos en cuanto a la secuenciación masiva han facilitado la aparición de nuevas aplicaciones en el diagnóstico genético clínico. Esto ha permitido aumentar la tasa diagnóstica, reducir el tiempo hasta obtener un diagnóstico y mejorar los tiempos de respuesta. Pero los avances tecnológicos no vienen carentes de desafíos. Las plataformas de alta procesabilidad generan gran cantidad de datos genómicos que suponen un reto para su interpretación. El procesamiento de los datos genómicos requiere de varios procesos bioinformáticos, pero es en el análisis terciario tras el *variant calling* y la anotación de las mismas donde se decide qué variante puede causar el fenotipo del paciente. Hace un tiempo, surgieron programas que ayudan a los profesionales que se enfrentan al filtrado, priorización, clasificación e interpretación de las variantes. Además de contener módulos de clasificación automática según las guías, integran algoritmos de IA para la priorización automática de variantes.

En este trabajo se presenta una primera evaluación retrospectiva con un grupo piloto de pacientes con el objetivo de establecer los criterios y validar la línea de trabajo posterior que pretende ayudar al especialista de laboratorio clínico a tener una visión global para seleccionar el programa de análisis terciario óptimo para su día a día.

La priorización de variantes es un paso crucial en el proceso de diagnóstico genético. Tradicionalmente, este proceso se realizaba de manera manual, aplicando una serie de filtros para reducir el número de variantes hasta identificar la potencial causa del fenotipo observado en el paciente. Este enfoque manual, aunque efectivo, demandaba un considerable tiempo y esfuerzo, ya que implicaba analizar un gran volumen de datos genómicos. Una vez filtradas las variantes, se procedía a su clasificación, tarea que también se realizaba manualmente siguiendo los criterios establecidos en las guías clínicas, como las del ACMG/AMP.

Desde una perspectiva de optimización de procesos, la priorización automática de variantes es fundamental para mejorar la eficiencia en el diagnóstico genético. No solo reduce significativamente el tiempo de respuesta, sino que también aumenta la capacidad de análisis de grandes volúmenes de datos genómicos, permitiendo una identificación más rápida y precisa de variantes relevantes para el diagnóstico clínico.

La clasificación automática representa un gran avance igualmente, pero queda relegada a un segundo plano. No todas las reglas se pueden automatizar, ya que algunas requieren información externa para su aplicación, como la herencia de la variante o la segregación de la enfermedad en la familia. Aunque en los últimos años se han realizado esfuerzos para estandarizar la aplicación de las reglas, sigue habiendo cierta subjetividad. Debido a esto, incluso la clasificación manual no está exenta de incertidumbre. Últimamente, para facilitar la clasificación estandarizada, están surgiendo artículos sugiriendo aproximaciones cuantitativas a la hora de aplicar las reglas [32], recomendaciones específicas a la hora de aplicar reglas concretas [33] y guías específicas de genes como, por el ejemplo las guías para el gen *APC* y los genes *BRCA1* y *BRCA2* [34], 35].

Centrándonos en los resultados del estudio, respecto a la priorización de variantes, SeqOne, CentoCloud y Franklin priorizaron todas las variantes y eVai dejó sin priorizar una única variante de las 24 evaluadas. En cuanto a Varsome Clinical su capacidad priorizadora es más que aceptable ya que colocó el 83 % de las variantes en el Top 5, pero no priorizó cuatro de 24 variantes siendo tres CNVs. Emedgene no priorizó una variante en el gen *PAX9*, lo cual resulta destacable, dado que esta variante tiene un carácter deletéreo. El cambio de nucleótido en la posición c.554, que transforma la citosina en adenina, genera un codón de parada prematuro. Esto probablemente conduce a la ausencia o disrupción de la proteína. Además, el fenotipo del paciente es muy específico y está bien documentado para este gen. Por otro lado, no detectó una deleción en el gen *NRXN1* situado en el cromosoma 2p16.3. Esta deleción de 2 exones pone de relieve las limitaciones intrínsecas del exoma en cuanto a la detección de CNVs de pequeño tamaño y la importancia de la generación del modelo de CNVs durante el análisis secundario. QCI Interpret, tiene un módulo de priorización potenciado por fenotipo (PDR del inglés phenotype driven ranking) que se cree que puede mejorar. Sorprende ver cómo en el listado general de variantes una variante en primera posición y clasificada como patogénica aplicando el PDR desaparece en el océano de variantes y la clasificación pasa a significado incierto. Se tiene la impresión de que su enfoque de priorización automática basado en fenotipos está demasiado relacionado con las patologías descritas, y esta rigidez no se alinea adecuadamente con el fenotipo del paciente. La evaluación del programa QCI Interpret para las 24 variantes se ha realizado en el listado general de las variantes sin utilizar la opción de PDR.

En cuanto a la clasificación automática, los programas Franklin, QCI Interpret y Varsome Clinical dieron mejor resultado, pero hay que resaltar que estos dos últimos no priorizaron un número considerable de variantes. En cambio, otros como CentoCloud, SeqOne y eVai, siendo más conservadores en su clasificación, priorizaron las variantes posicionándolas en los primeros puestos en el ranking, pero manteniendo la clasificación como de significado incierto a la espera de soporte clínico externo.

En resumen, se considera que no hay un programa perfecto; sin embargo, el programa óptimo deberá ser aquel capaz de priorizar automáticamente al máximo sin perder ninguna variante causal. La clasificación automática deberá ser, además, precisa. Los datos expuestos en esta comparativa demuestran la gran capacidad de priorización de los programas gracias a los algoritmos bioinformáticos y de IA.

Estos programas han demostrado su potencial de manera global, mostrando una notable capacidad para priorizar y clasificar variantes de forma automática. Sin embargo, existen diferencias que destacan la importancia de una priorización adecuada para alcanzar un diagnóstico exitoso en los pacientes. La clasificación automática, aunque es un valor añadido significativo de estos programas, puede ser modificada y/o cuestionada por otra información decisiva que tanto los analistas como los clínicos puedan tener a su disposición. Es evidente que estos programas bioinformáticos están en constante evolución, mejorando sus capacidades para mantenerse al día con los avances científicos. En general, los programas evaluados no solo apoyan, sino que también optimizan las tareas de los especialistas en laboratorio clínico, facilitando así la implementación del WES en la práctica clínica.

Los resultados presentados tienen la limitación de basarse en un número reducido de variantes. Para obtener resultados más consistentes, sería valioso ampliar el estudio no solo a variantes patogénicas y probablemente patogénicas, sino también incluir una selección de variantes poblacionales. Esto nos permitiría evaluar mejor la capacidad de genotipado y de clasificación automática de cada programa.

En conclusión, el programa Franklin ha demostrado un rendimiento global superior. En lo que respecta a la priorización automática, se ha situado en tercer lugar, detrás de SeqOne y CentoCloud. En términos de clasificación automática, Franklin ha alcanzado un mayor porcentaje de concordancia con la clasificación de referencia y ha presentado menos discordancias con implicaciones clínicas.

## Supplementary Material

Supplementary Material

## Material Suplementario

Este artículo incluye material suplementario (https://doi.org/10.1515/almed-2024-0101).
