# Supplementary material for: Evaluación de siete programas bioinformáticos para el análisis terciario de datos genómicos generados a partir de la secuenciación del exoma completo en un grupo piloto de pacientes
Source: Adv Lab Med. 2025 Feb 10;6(1):37–45. [Article in Spanish] doi: 10.1515/almed-2024-0101 (PMC11949555; doi:10.1515/almed-2024-0101)
Supplement: Supplementary file 1 — Supplementary Material [file j_almed-2024-0101_suppl_001.docx]

**Material Suplementario Tablas 1-7.**

**Abreviaturas:**

ACMG, American College of Medical Genetics and Genomics

AD, autosómico dominante

AMP, Association for Molecular Pathology

AR, autosómico recesivo

B, benigno

BP, break point

C, contiguos

Clas, clasificación

CNV, copy number variant- cambio de número de copias

Del, deleción

Dup, duplicación

F, femenino

Het, heterocigoto

Hom, homocigoto

HPO, Human Phenotype Ontology

Idic, isodicéntrico

Inv, inversión

kb, kilobase

LXD, ligado al cromosoma X dominante

m, moderate

M, masculino

NC, no contiguo

ND, no detectado

NP, no priorizado

OMIM, Online Mendelian Inherited in Man

P, patogénico

PP, probablemente patogénico

Pos, posición

QCII, Qiagen Clinical Insight Interpret

Ref, referencia

sp, del inglés supporting

st, del inglés strong

V, variante de significado incierto

vst, del inglés very strong

**Tabla 1.** Clasificación de referencia de las CNVs aplicando los criterios de las guías clínicas.

| Paciente | Localización Cromosómica | Coordenadas genómicas GRCh38 | Tipo de CNV | Tamaño kb | Clas  Ref | Criterios ACMG/ClinGen |
| --- | --- | --- | --- | --- | --- | --- |
| R1 | 2p16.3 | NC_000002.12:g.(51026361_51028284)del | Del | 1,9 | P | 2C(+0,9),4L(+0,15) |
| R4 | 2q11.1q11.2 | NC_000002.12:g.(95948520_96986324)del | Del | 1040 | PP | 2A(+0,9) |
|  | 15q11.2 | NC_000015.10:g.(22786647_23039554)del | Del | 253 | PP | 2A(+0,9) |
| R9 | Xp22.31 | NC_000023.11:g.(7049993_7350563)del | Del | 300 | P | 2A(+1) |
| R11 | 15q11.2q13.3 | NC_000015.10:g.(22786647_32158617)dup | Dup | 9370 | P | 2A(+1),3B(+0,45),4L(+0,15) |
| R20 | 11q24.2q25 | NC_000011.10:g.(127000445_134387705)del | Del | 7390 | P | 2A(+1),3B(+0,45),4L(+0,15) |

**Tabla 2.** Clasificación de referencia de los SNVs y deleciones y duplicaciones pequeñas aplicando los criterios de las guías clínicas.

| Paciente | Gen | Trascrito MANE Select | Variante | Efecto proteico | Tipo de variante | Cigosidad | Clas  Ref | Criterios ACMG/AMP |
| --- | --- | --- | --- | --- | --- | --- | --- | --- |
| R1 | *SPAST* | NM_014946.4 | c.1617-2A>G | - | Splicing | Het | P | PVS1(vst);PM2(sp);PP5(sp) |
| R2 | *SPAST* | NM_014946.4 | c.1379G>A | p.Arg460His | Missense | Het | P | PM1(st);PM2(sp);PM5(m);PP3(sp);PP5(sp) |
| R3 | *SPG11* | NM_025137.4 | c.6832_6833del | p.Ser2278Leufs*61 | Frameshift | Hom | P | PVS1(vst);PM2(sp);PP1(sp);PP5(sp) |
| R5 | *CUBN* | NM_001081.4 | c.9524C>A | p.Ser3175* | Nonsense | Hom | PP | PVS1(vst);PM2(sp) |
| R6 | *CLCN1* | NM_000083.3 | c.742A>T | p.Lys248* | Nonsense | Het | P | PVS1(vst);PM2(sp);PM3(sp);PP5(sp) |
|  |  |  | c.2363A>C | p.Gln788Pro | Missense | Het | PP | PS3(st);PM2(sp);PM3(sp);PP5(sp) |
| R7 | *SLC3A1* | NM_000341.4 | c.1011G>A | p.Pro337= | Silent | Het | PP | PS3(st);PM2(sp);PM3(sp);PP5(sp) |
|  |  |  | c.1354C>T | p.Arg452Trp | Missense | Het | P | PM1(st);PM2(sp);PM3(sp);PM5(m);PP3(sp);PP5(sp) |
| R8 | *EXT1* | NM_000127.3 | c.1037G>T | p.Arg346Ile | Missense | Het | PP | PM2(sp);PM5(m);PP3(sp);PP5(m) |
| R10 | *SACS* | NM_014363.6 | c.5115_5116del | p.Ser1706Phefs*9 | Frameshift | Hom | PP | PVS1(vst);PM2(sp) |
| R12 | *CNOT1* | NM_016284.5 | c.2071del | p.Val691Leufs*13 | Frameshift | Het | PP | PVS1(vst);PM2(sp) |
| R13 | *NSD1* | NM_022455.5 | c.4467del | p.Asp1489Glufs*14 | Frameshift | Het | PP | PVS1(vst);PM2(sp) |
| R14 | *PAX9* | NM_001372076.1 | c.554C>A | p.Ser185* | Nonsense | Het | PP | PVS1(vst);PM2(sp) |
| R15 | *CDKL5* | NM_001323289.2 | c.283-2A>G | - | Splicing | Het | PP | PVS1(vst);PM2(sp) |
| R16 | *SGCE* | NM_003919.3 | c.884dup | p.Leu295Phefs*3 | Frameshift | Het | P | PVS1(vst);PM2(sp);PP5(sp) |
| R17 | *BSND* | NM_057176.3 | c.23G>A | p.Arg8Gln | Missense | Hom | PP | PM1(st);PM2(sp);PM5(m);PP3(sp);PP5(sp) |
| R18 | *EXT2* | NM_207122.2 | c.514C>T | p.Gln172* | Nonsense | Het | P | PVS1(vst);PM2(sp);PP5(sp) |
| R19 | *JAG1* | NM_000214.3 | c.221_224del | p.Tyr74Serfs*86 | Frameshift | Het | PP | PVS1(vst);PM2(sp) |

**Tabla 3.** Pacientes con CNVs**.**

| Paciente | Localización Cromosómica | Coordenadas genómicas GRCh38 | Tipo de CNV | Número de copias | Tamaño (kb) | Clas  Ref | ClinGen |
| --- | --- | --- | --- | --- | --- | --- | --- |
| R1 | 2p16.3 | NC_000002.12:g.(51026361_51028284)del | Deleción | 1 | 1,9 | P | Deleción intragénica gen *NRXN1*: trastorno complejo del neurodesarrollo |
| R4 | 2q11.1q11.2 | NC_000002.12:g.(95948520_96986324)del | Deleción | 1 | 1040 | PP | Deleción recurrente 2q11.2 |
|  | 15q11.2 | NC_000015.10:g.(22786647_23039554)del | Deleción | 1 | 253 | PP | Deleción recurrente 15q11.2 (BP1-BP2) |
| R9 | Xp22.31 | NC_000023.11:g.(7049993_7350563)del | Deleción | 0 | 300 | P | Deleción recurrente Xp22.31 que incluye el gen *STS* asociado a Ictiosis ligada a X recesiva |
| R11 | 15q11.2q13.3 | NC_000015.10:g.(22786647_32158617)dup | Duplicación | 4 | 9370 | P | Tetrasomías del cromosoma 15 con alta sospecha de cromosoma marcador inv dup(15 ) o idic(15) |
| R20 | 11q24.2q25 | NC_000011.10:g.(127000445_134387705)del | Deleción | 1 | 7390 | P | Región terminal 11q23q25 asociado al Síndrome Jacobsen |

**Tabla 4.** Pacientes con SNVs y deleciones y duplicaciones pequeñas.

| Paciente | Gen | Trascrito MANE Select | Coordenada genómica GRCh38 | Variante | Efecto proteico | Tipo de variante | Herencia | Cigosidad | Clas  Ref | OMIM | Enfermedad |
| --- | --- | --- | --- | --- | --- | --- | --- | --- | --- | --- | --- |
| R1 | *SPAST* | NM_014946.4 | 2:32144935 | c.1617-2A>G | - | Splicing | AD | Het | P | #182601 | Paraplejia espástica 4, autosómica dominante |
| R2 | *SPAST* | NM_014946.4 | 2:32136934 | c.1379G>A | p.Arg460His | Missense | AD | Het | P | #182601 | Paraplejia espástica 4, autosómica dominante |
| R3 | *SPG11* | NM_025137.4 | 15:44566227 | c.6832_6833del | p.Ser2278Leufs*61 | Frameshift | AR | Hom | P | #604360 | Paraplejia espástica 11, autosómica recesiva |
| R5 | *CUBN* | NM_001081.4 | 10:16851374 | c.9524C>A | p.Ser3175* | Nonsense | AR | Hom | PP | #261100 | Síndrome de Imerslund-Grasbeck 1 |
| R6 | *CLCN1* | NM_000083.3 | 7:143323354 | c.742A>T | p.Lys248* | Nonsense | AR | Het | P | #255700 | Miotonía congénita autosómica recesiva |
|  |  |  | 7:143346657 | c.2363A>C | p.Gln788Pro | Missense |  | Het | PP |  |  |
| R7 | *SLC3A1* | NM_000341.4 | 2:44300090 | c.1011G>A | p.Pro337= | Silent | AR | Het | PP | #220100 | Cistinuria |
|  |  |  | 2:44312607 | c.1354C>T | p.Arg452Trp | Missense |  | Het | P |  |  |
| R8 | *EXT1* | NM_000127.3 | 8:117837127 | c.1037G>T | p.Arg346Ile | Missense | AD | Het | PP | #133700 | Exostosis múltiples tipo 1 |
| R10 | *SACS* | NM_014363.6 | 13:23338760 | c.5115_5116del | p.Ser1706Phefs*9 | Frameshift | AR | Hom | PP | #270550 | Ataxia espástica tipo Charlevoix-Saguenay |
| R12 | *CNOT1* | NM_016284.5 | 16:58560271 | c.2071del | p.Val691Leufs*13 | Frameshift | AD | Het | PP | **#**619033 | Síndrome de Vissers-Bodmer |
| R13 | *NSD1* | NM_022455.5 | 5:177246766 | c.4467del | p.Asp1489Glufs*14 | Frameshift | AD | Het | PP | #117550 | Síndrome de Sotos |
| R14 | *PAX9* | NM_001372076.1 | 14:36663446 | c.554C>A | p.Ser185* | Nonsense | AD | Het | PP | #604625 | Agenesia dental selectiva 3 |
| R15 | *CDKL5* | NM_001323289.2 | X:18579846 | c.283-2A>G | - | Splicing | LXD | Het | PP | #300672 | Encefalopatía epiléptica y del desarrollo 2 |
| R16 | *SGCE* | NM_003919.3 | 7:94600798 | c.884dup | p.Leu295Phefs*3 | Frameshift | AD | Het | P | #159900 | Distonía mioclónica 11 |
| R17 | *BSND* | NM_057176.3 | 1:54999209 | c.23G>A | p.Arg8Gln | Missense | AR | Hom | PP | #602522 | Sordera neurosensorial con disfunción renal leve |
| R18 | *EXT2* | NM_207122.2 | 11:44108226 | c.514C>T | p.Gln172* | Nonsense | AD | Het | P | #133701 | Exostosis múltiples tipo 2 |
| R19 | *JAG1* | NM_000214.3 | 20:10672864 | c.221_224del | p.Tyr74Serfs*86 | Frameshift | AD | Het | PP | #118450 | Síndrome Alagille 1 |

**Tabla 5.** Fenotipo de cada paciente en términos HPO.

| Paciente | Género | Términos HPO |
| --- | --- | --- |
| R1 | M | Global developmental delay HP:0001263; Delayed speech and language development HP:0000750; Drooling HP:0002307; Affected HP:0032320 |
| R2 | M | Gait disturbance HP:0001288; Spastic paraparetic gait HP:0031958; Intermittent painful muscle spasms HP:0011964; Affected HP:0032320 |
| R3 | F | Paraparesis HP:0002385; Cognitive impairment HP:0100543; Hypertelorism HP:0000316; Wide nasal bridge HP:0000431; Prominent nasal bridge HP:0000426; Specific learning disability HP:0001328; Affected HP:0032320 |
| R4 | M | Tall stature HP:0000098; Neurodevelopmental delay HP:0012758; Nevus of Ota HP:0009920; Unilateral cryptorchidism HP:0012741; Hypotonia HP:0001252; Autistic behavior HP:0000729; Affected HP:0032320 |
| R5 | M | Proteinuria HP:0000093; Abnormal urine protein level HP:0020129; Glomerular proteinuria HP:4000058; Affected HP:0032320 |
| R6 | M | Myotonia HP:0002486; Exercise-induced muscle stiffness HP:0008967; Myotonia of the upper limb HP:0012903; Myotonia of the lower limb HP:0012902; Affected HP:0032320 |
| R7 | F | Cystinuria HP:0003131; Increased sulfur amino acid level in urine HP:0033095; Aminoaciduria HP:0003355; Affected HP:0032320 |
| R8 | M | Short stature HP:0004322; Genu valgum HP:0002857; Osteochondrosis HP:0040188; Chronic kidney disease HP:0012622; Affected HP:0032320 |
| R9 | M | Ichthyosis HP:0008064; Dry skin HP:0000958; Scaling skin HP:0040189; Affected HP:0032320 |
| R10 | F | Dystonia HP:0001332; Chorea HP:0002072; Spasticity HP:0001257; Tip-toe gait HP:0030051; Clumsiness HP:0002312; Dysmetria HP:0001310; Babinski sign HP:0003487; Hyperreflexia HP:0001347; Clonus HP:0002169; Unsteady gait HP:0002317 Affected HP:0032320 |
| R11 | M | Infantile axial hypotonia HP:0009062; Limb hypertonia HP:0002509; Neurodevelopmental delay HP:0012758; Affected HP:0032320 |
| R12 | F | Intellectual disability HP:0001249; Coloboma HP:0000589; Esotropia HP:0000565; Specific learning disability HP:0001328; Abnormal social behavior HP:0012433; Language impairment HP:0002463; Affected HP:0032320 |
| R13 | F | Retrognathia HP:0000278; 2-3 finger syndactyly HP:0001233; Short neck HP:0000470; Microphthalmia HP:0000568; Narrow mouth HP:0000160; Global developmental delay HP:0001263; Atypical behavior HP:0000708; Micrognathia HP:0000347 Wide nasal bridge HP:0000431; Epicanthus HP:0000286; Long philtrum HP:0000343; Aggressive behavior HP:0000718; Tall stature HP:0000098; Recurrent respiratory infections HP:0002205; Affected HP:0032320 |
| R14 | M | Low-set ears HP:0000369; Agenesis of incisor HP:0006485; Protruding ear HP:0000411; Affected HP:0032320 |
| R15 | F | Neurodevelopmental delay HP:0012758; Hypotonia HP:0001252; Seizure HP:0001250; Affected HP:0032320 |
| R16 | F | Gait disturbance HP:0001288; Myoclonus HP:0001336; Delayed speech and language development HP:0000750; Language impairment HP:0002463; Dystonia HP:0001332; Hypertonia HP:0001276; Affected HP:0032320 |
| R17 | F | Retrognathia HP:0000278; Impulsivity HP:0100710; Microphthalmia HP:0000568; Astigmatism HP:0000483; Atypical behavior HP:0000708; Hypermetropia HP:0000540; Sensorineural hearing impairment HP:0000407; Microcephaly HP:0000252 Intellectual disability HP:0001249; Affected HP:0032320 |
| R18 | F | Multiple exostoses HP:0002762; Multiple enchondromatosis HP:0005701; Osteochondroma HP:0030431; Affected HP:0032320 |
| R19 | M | Mandibular prognathia HP:0000303; Partial anomalous pulmonary venous return HP:0010773; Butterfly vertebrae HP:0003316; Prominent forehead HP:0011220; Triangular face HP:0000325; Cholestasis HP:0001396; Small for gestational age HP:0001518; Intrauterine growth retardation HP:0001511; Patent foramen ovale HP:0001655; Abnormal calvaria morphology HP:0002683; Jaundice HP:0000952; Affected HP:0032320 |
| R20 | F | Intellectual disability HP:0001249; Gait disturbance HP:0001288; Broad-based gait HP:0002136; Frequent falls HP:0002359; Upper motor neuron dysfunction HP:0002493; Seizure HP:0001250; Hypomimic face HP:0000338; Camptocormia HP:0100595; Parkinsonism HP:0001300; Rigidity HP:0002063; Asterixis HP:0012164; Ataxia HP:0001251; Brisk reflexes HP:0001348; Impaired vibratory sensation HP:0002495; Bradykinesia HP:0002067; Lower limb muscle weakness HP:0007340; Dysmetria HP:0001310; Hypointensity of cerebral white matter on MRI HP:0007103; Abnormal cerebral vascular morphology HP:0100659; Leukoencephalopathy HP:0002352; Cranial hyperostosis HP:0004437; Affected HP:0032320 |

| Paciente | Localización Cromosómica | Tipo de CNV | Tamaño kb | Clas  Ref | Emedgene | | eVai | | Varsome Clinical | | Centocloud | | QCII | | SeqOne | | Franklin | |
| --- | --- | --- | --- | --- | --- | --- | --- | --- | --- | --- | --- | --- | --- | --- | --- | --- | --- | --- |
|  |  |  |  |  | Clas | Pos | Clas | Pos | Clas | Pos | Clas | Pos | Clas | Pos | Clas | Pos | Clas | Pos |
| R1 | 2p16.3 | Del | 1,9 | P | - | ND | PP | 5 | V | NP | PP | 1 | PP | NP | PP | 1 | P | 10 |
| R4 | 2q11.1q11.2 | Del | 1040 | PP | P | 10 | P | 5 | P | 1 | P | 1 | P | 1 | P | 1 | P | 10 |
|  | 15q11.2 | Del | 253 | PP | P | 1 | P | 5 | B | NP | B | 10 | PP | 5 | V | 5 | P | 10 |
| R9 | Xp22.31 | Del | 300 | P | P | 1 | P | 10 | V | NP | P | 1 | P | 1 | PP | 1 | P | 5 |
| R11 | 15q11.2q13.3 | Dup | 9370 | P | P | 1 | P | 1 | P | 1 | P | 1 | V | 1 | V | 1 | P | 5 |
| R20 | 11q24.2q25 | Del | 7390 | P | V | 1 | P | 5 | P | 1 | P | 1 | P | 1 | V | 1 | P | 10 |

**Tabla 6.** Comparativa entre los programas en la clasificación y posición en la priorización en pacientes con CNVs.

| Paciente | Gen | Variante | Efecto proteico | Clas Ref | Emedgene | | eVai | | Varsome Clinical | | Centocloud | | QCII | | SeqOne | | Franklin | | |
| --- | --- | --- | --- | --- | --- | --- | --- | --- | --- | --- | --- | --- | --- | --- | --- | --- | --- | --- | --- |
|  |  |  |  |  | Clas | Pos | Clas | Pos | Clas | Pos | Clas | Pos | Clas | Pos | Clas | Pos | Clas | Pos |  |
| R1 | *SPAST* | c.1617-2A>G | - | P | P | 1 | P | 1 | P | NP | PP | 5 | P | 1 | P | 1 | P | 10 |  |
| R2 | *SPAST* | c.1379G>A | p.Arg460His | P | P | 5 | P | 1 | P | 1 | V | 1 | PP | 1 | PP | 1 | P | 1 |  |
| R3 | *SPG11* | c.6832_6833del | p.Ser2278Leufs*61 | P | P | 1 | P | 1 | P | 1 | P | 1 | P | 1 | P | 1 | P | 1 |  |
| R5 | *CUBN* | c.9524C>A | p.Ser3175* | PP | PP | 1 | V | 1 | PP | 1 | V | 1 | P | 10 | PP | 1 | P | 1 |  |
| R6 | *CLCN1* | c.742A>T | p.Lys248* | P | P | 5 | P | 5 | P | 1 | P | 5 | P | 5 | P | 1 | P | 5 |  |
|  |  | c.2363A>C | p.Gln788Pro | PP | V | 1 (C) | V | 5 (NC) | PP | 5 (C) | PP | 5 (C) | V | 10 (C) | V | 5 (C) | PP | 1 (C) |  |
| R7 | *SLC3A1* | c.1011G>A | p.Pro337= | PP | V | 1 | V | 5 | P | 5 | V | 5 | P | 5 | V | 5 | V | 5 |  |
|  |  | c.1354C>T | p.Arg452Trp | P | PP | 5 (C) | V | 5 (C) | P | 1 (C) | PP | 5 (C) | P | 1 (C) | PP | 1 (NC) | P | 1 (C) |  |
| R8 | *EXT1* | c.1037G>T | p.Arg346Ile | PP | PP | 1 | PP | 1 | P | 1 | V | 1 | V | NP | PP | 1 | P | 1 |  |
| R10 | *SACS* | c.5115_5116del | p.Ser1706Phefs*9 | PP | PP | 1 | P | 5 | PP | 1 | V | 1 | PP | 15 | PP | 1 | PP | 1 |  |
| R12 | *CNOT1* | c.2071del | p.Val691Leufs*13 | PP | PP | 1 | P | 1 | PP | 5 | V | 1 | PP | NP | PP | 5 | PP | 1 |  |
| R13 | *NSD1* | c.4467del | p.Asp1489Glufs*14 | PP | PP | 1 | PP | 1 | PP | 1 | PP | 1 | PP | 10 | PP | 1 | PP | 1 |  |
| R14 | *PAX9* | c.554C>A | p.Ser185* | PP | P | NP | P | 1 | P | 1 | V | 5 | PP | NP | P | 1 | PP | 5 |  |
| R15 | *CDKL5* | c.283-2A>G | - | PP | P | 1 | P | 1 | PP | 5 | PP | 1 | - | ND | PP | 1 | PP | 5 |  |
| R16 | *SGCE* | c.884dup | p.Leu295Phefs*3 | P | PP | 1 | P | 1 | P | 5 | PP | 1 | P | 1 | P | 1 | P | 1 |  |
| R17 | *BSND* | c.23G>A | p.Arg8Gln | PP | V | 10 | V | NP | P | 1 | V | 10 | PP | NP | V | 15 | V | 5 |  |
| R18 | *EXT2* | c.514C>T | p.Gln172* | P | P | 1 | P | 1 | P | 1 | P | 1 | P | 5 | P | 1 | P | 1 |  |
| R19 | *JAG1* | c.221_224del | p.Tyr74Serfs*86 | PP | PP | 1 | P | 1 | PP | 5 | PP | 1 | PP | NP | PP | 1 | PP | 1 |  |

**Tabla 7.** Comparativa entre los programas en la clasificación y posición en la priorización en pacientes SNVs y deleciones y duplicaciones pequeñas.
